# Experiment and Analysis of Temperature Sensing of Microstructured Fiber with Silver and PDMS Films

**DOI:** 10.3390/s22041447

**Published:** 2022-02-13

**Authors:** Shuguang Li, Song Zhang, Ying Guo, Hongyu Li, Yujun Wang, Xue Zhou, Tonglei Cheng

**Affiliations:** 1 State Key Laboratory of Metastable Materials Science & Technology, School of Science, Yanshan University, Qinhuangdao 066004, China; zhangsong0712@163.com (S.Z.); guoying@stumail.ysu.edu.cn (Y.G.); hongyu0603@163.com (H.L.); yujunwangsn@stumail.ysu.edu.cn (Y.W.); 2 The State Key Laboratory of Synthetical Automation for Process Industries, The College of Information Science and Engineering, Northeastern University, Shenyang 110819, China; zhouxue@ise.neu.edu.cn (X.Z.); chengtonglei@ise.neu.edu.cn (T.C.)

**Keywords:** microstructure fiber, temperature sensor, surface plasmon resonance, silver mirror reaction, polydimethylsiloxane (PDMS)

## Abstract

In this study, the silver mirror reaction was used to coat the silver film on the surface of self-made microstructured fiber (MSF) to stimulate the surface plasmon resonance effect, and Polydimethylsiloxane (PDMS) with a high thermal-optical coefficient was coated on the silver film as temperature-sensitive material. The MSF with silver and PDMS films was coupled with multi-mode fiber on both sides to form the temperature sensor. In this sensor system, the energy is coupled into the cladding of the microstructure fiber by multi-mode fiber, and the surface plasmon resonance can be further excitated in the MSF. When the temperature of the external environment changes, the refractive index of PDMS will also change. At this time, combined with the surface plasmon resonance effect, a resonant absorption peak corresponding to the temperature appears in the transmission spectrum so that the temperature can be measured quickly and accurately. We found that, in the temperature range of 35 °C to 95 °C, the average temperature sensitivity of the sensor during heating and cooling was −0.83 nm/°C and −0.84 nm/°C, respectively. The advantages of this sensor are the simple structure, convenient operation and good reversibility. The relative sensitivity deviation value (RSD = 0.0059) showed that the sensor has high stability. The temperature sensor based on MSF has favorable prospects for use in fields such as medical treatment, biochemical detection and intelligent monitoring.

## 1. Introduction

Temperature is a physical quantity used to measure the degree of heat and cold of materials. It can only be indirectly measured based on the characteristics of the object changing with temperature. Therefore, accurate temperature measurement and control is highly important in many fields, such as chemical reaction, biomedical and environmental monitoring [1,2]. Microstructured fiber (MSF) has become a popular type of sensing platform due to its unique light-guiding properties and designable structure. In recent years, temperature sensors based on MSF with different measurement principles or structures have been proposed. Compared with the traditional sensor, the MSF sensor has the advantages of a small volume, light weight, fast reaction speed, strong anti-electromagnetic interference ability and chemical corrosion resistance, among other benefits [3,4,5]. It can be used to investigate temperature detection in many special environments and has been widely applied and studied in recent years.

Surface plasmon resonance is an optical phenomenon that occurs at the interface between the medium and the metal [6]. When light is incident from the optically dense medium to the optically thin medium at a certain range of angles, total reflection will occur. However, there are still certain electromagnetic waves (evanescent waves) in the optically thin medium that penetrate the metal film during this process and excite free electrons to oscillate on its surface. When the incident angle and the wavelength meet the wave mismatch condition, the frequency of the incident light and the evanescent wave is equal, and the photon and the free electrons on the metal surface couple and resonate; that is, surface plasmon resonance occurs. Subsequently, we apply the principle of surface plasmon to the optical fiber, which replaces the prism in the prism coupling structure of optical fiber [7]. As a result, a macroscopically reflected phenomenon, namely a resonance absorption peak, appears in the transmission spectrum. In addition, there is a corresponding relationship between the location of the absorption peak and the refractive index of the metal surface medium [8,9,10]. Therefore, we can infer the refractive index of the measured object in the external environment from the change of the resonance absorption peak position in the transmission spectrum. Moreover, we can deduce other physical parameters derived from the refractive index, including temperature variables that will be studied in this paper. This is the basic principle of fiber optic plasmonic sensing.

In 2016, Liu Chao et al. designed a temperature sensor with an MSF structure based on the SPR effect. As a result of the coupling principle between the plasmon mode and the fiber core fundamental mode, the temperature sensitivity was 3080 pm/°C and the resolution reached 0.01325 °C [11]. In 2017, Yang et al. investigated a fiber optic ring laser temperature sensor with a high refractive index liquid filled with MSF, and the temperature sensitivity of the wavelength modulation and intensity modulation reached −1.747 nm/°C and 0.137 dB/°C, respectively [12]. Du et al. investigated a microstructure long-period fiber grating (MSF-LPG) filled with isopropyl alcohol, which could realize high temperature sensitivity sensing at 1.356 nm/°C, within a range of 20–50 °C [13]. Pang et al. proposed an MSF temperature sensor with a temperature-sensitive material silicone coating the fiber cladding, and the sensitivity was 240 pm/°C from −20 °C to 80 °C [14]. In 2016, Lu et al. proposed a silver-plated SPR sensor inside a hollow fiber that was filled with liquid crystal for temperature sensing. In the 20–34.5 °C temperature range, the temperature sensitivity reached 4.72 nm/°C; however, due to the unique properties of liquid crystals, the temperature sensitivity decreased to 0.55 nm/°C in 36–50 °C temperatures [15].

Temperature measurement is mainly conducted by filling air holes with liquid temperature-sensitive materials to form temperature sensing channels. The temperature-sensitive materials, such as benzene and alcohol, are not only toxic but also prone to leakage or evaporation. Moreover, their reversibility and operability are poor. Additionally, liquid crystal narrows the sensor temperature range due to its unstable physical properties.

At present, many high thermal optical coefficient materials have been applied to fiber temperature sensor research. Based on the above, PDMS was proposed as a temperature-sensitive substance [16]. PDMS is a type of high molecular compound in thermal optical materials, which is usually called an organosilicon. It is non-toxic and tasteless, non-volatile, possesses adequate chemical stability and light transmittance, is straightforward to connect with a variety of materials, and among other advantages [16], it can be employed at −50 °C to 200 °C temperatures for long time periods. Compared to other thermal sensitive media, the thermal optical coefficient of PDMS (γ = −4.66 × 10^−4^/°C) is much higher [17]. The refractive index of PDMS at different temperatures is defined as n_T_, and the refractive index is 1.4204 at 22 °C. According to n_T_ = n_0_ + γ × ΔT, the n_T_ decreases when temperatures increase [18,19]. Based on the modal interference in PDMS/silica hybrid fiber structures, Gao et al. proposed a high sensitivity temperature sensor duo with a large thermo-optical coefficient [20]. An optical fiber embedded in PDMS based on SPR was reported by Velazquez-Gonzalez et al.; it acts as a temperature to refractive index transducer. This sensor is appealing for temperature monitoring in microfluidic devices made of PDMS due to its high-quality performance and simple fabrication process [21]. 

The fabrication cost, temperature sensitivity, measurement range and stability of fiber temperature sensors are important indexes to judge a sensor’s performance [22]. Therefore, we used a self-made microstructured fiber with silver and PDMS films as the sensitive head for the temperature sensor in this paper. This type of microstructural fiber has a small core diameter, few porosity layers and an easy-to-excite cladding mode. An MSF sensor was fabricated by coating the metal film to excite the SPR phenomenon and using PDMS with a high thermal optic coefficient. The stability of this sensor was enhanced by adding the PDMS, which provides a protective barrier for the thin silver layer against dust and oxidation. The average temperature sensitivities of the sensor were −0.83 nm/°C and −0.84 nm/°C during the heating and cooling processes, respectively, in a 35 °C to 95 °C temperature range. It may have broad application prospects to developments in the medical and electronic industries. 

## 2. Experimental Principle and Fabrication Process

**Figure 1:**
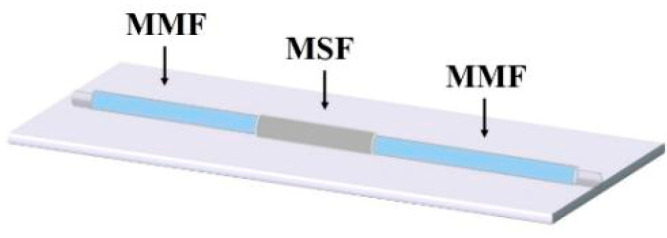
The sensor structure of MMF-MSF-MMF.

The structure (shown in Figure 1) of the sensing model constructed in this experiment was a multimode fiber (MMF)-microstructure fiber (MSF)-multimode fiber. The MMF sections on both sides of the structure were a standard size; the core diameter and cladding diameters of the MMF sections were 62.5 μm and 125 μm, respectively. 

**Figure 2:**
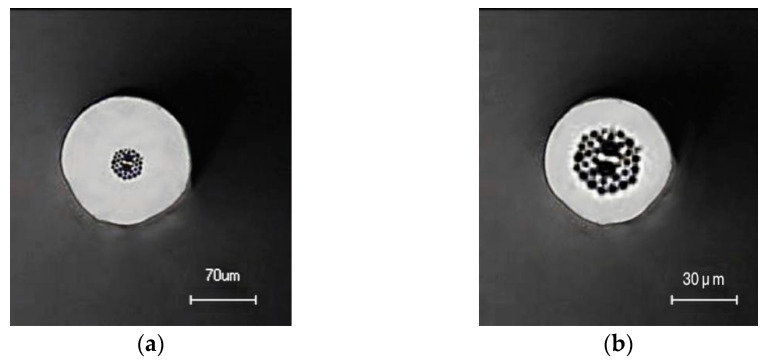
(**a**) The cross-section of the MSF; (**b**) the cross-section of the MSF after corrosion treatment.

The fabrication process of the microstructured fiber used in our experiment was as follows: firstly, the prefabricated rod was prepared by means of a stacking method using a hollow glass tube and solid glass rod. The outer diameter of the prefabricated rod was about 20 mm. To preserve the air holes in the MSF during the drawing process, the MSF was prepared using the two-step drawing method. The outer diameter of the prefabricated rod was about 3.0 mm after the first step drawing, and an MSF with an outer diameter of 140 μm was obtained following the second step drawing. Among them, the cross-sectional size of the microstructure fiber as the core area of the sensing was as follows: the diameter of the air hole cladding was about 35 μm, and the total outer diameter was 140 μm, as seen in Figure 2a. Figure 2a illustrates that in order to prevent the cladding from being too thick to affect the sensing, we corroded the fiber using hydrofluoric acid, etching it to an outer diameter of about 70 μm in Figure 2b. 

### 2.1. Silver Plating by Silver Mirror Reaction

To produce the SPR effect, this portion of the preparation experiment involved using the silver mirror reaction method to coat the silver film on the outer surface of the microstructured fiber. The silver mirror reaction (Torrance reaction) uses a reducing agent (such as glucose) to reduce the silver ammonia complex to metallic silver nanoparticles. The detailed reaction equations of the silver mirror reaction are as follows [23]:

(1) $$CH_{2}OH{\left(CHOH\right)}_{4}COH+2Ag{\left(NH_{a}\right)}_{2}^{+}+2OH^{-}\to 2Ag+\;CH_{2}OH{\left(CHOH\right)}_{4}COOH+4NH_{a}^{+}+H_{2}O$$

(2) $$2AgNO_{a}+2KOH\to Ag_{2}O+2KNO_{a}+H_{2}O$$

(3) $$Ag_{2}O+4NH_{a}+2KNO_{a}+H_{2}O\to 2Ag{\left(NH_{a}\right)}_{a}NO_{a}+2KOH\;$$

First, a certain proportion of silver nitrate solution (AgNO_3_) and potassium hydroxide solution (KOH) were mixed in a magnetic stirrer to produce a unique brown precipitate, and then ammonia water (NH_3_·H_2_O) was added until the solution became transparent to obtain the silver ammonium complex. Glucose was then used to reduce the silver ammonia complex to metallic silver nanoparticles. We dropped the prepared liquid on the position where the optical fiber needed to be coated, and then a uniform silver film can be coated on the outer surface of the optical fiber.

**Figure 3:**
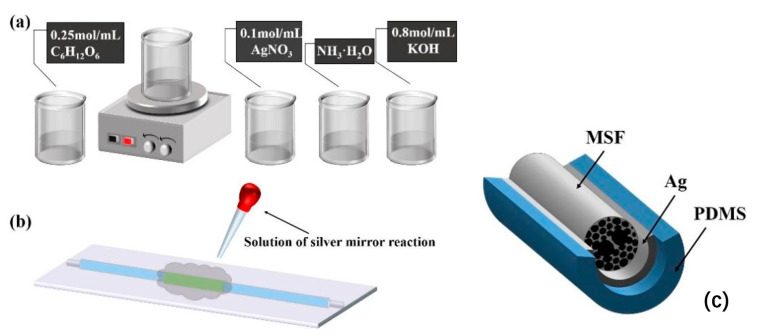
(**a**) Configuration process of silver ammonium complex; (**b**) coating the MSF with Ag thin layer; (**c**) the three-dimensional structure of the MSF coated with silver film and PDMS.

By changing the concentration of the silver ammonium complex and reaction time of the silver mirror reaction, the distribution density and particle size of silver nanoparticles on the surface of fibers can be affected [24]. The main purpose of this experiment was not to explore the effect of silver film thickness on the sensing effect. Under the existing conditions in the laboratory, we chose to control the time and concentration ratio of the silver mirror reaction to achieve surface plasmon resonance. Although this method could not accurately determine the thickness of the silver film, it ensured the appearance of the surface plasmon resonance effect in the sensing experiment. Therefore, in this test process, the experiment was configured according to a fixed concentration ratio, specifically: 0.1 mol/mL silver nitrate solution (AgNO_3_), 0.8 mol/mL potassium hydroxide solution (KOH) and 0.25 mol/mL reducing agent glucose solution (C_6_H_12_O_6_) to produce the silver mirror reaction in a 5:7:5 volume ratio (see Figure 3a,b). 

The reaction solution was dropped on the sensing part in an extremely short time. After 20 min of reaction time, the surface of the MSF was washed with deionized water to complete the silver plating. See Figure 3b for the schematic diagram.

The temperature-sensitive material PDMS was coated to enhance its temperature-sensitive characteristics, as shown in Figure 3c. To demonstrate the structure of the MSF more clearly and aesthetically, the air holes part of the MSF cladding is displayed in the three-dimensional structure diagram in Figure 3c. The specific experimental methods and material properties will be introduced below. 

### 2.2. Manufacturing Process of PDMS-Wrapped MSF

PDMS is a type of transparent material that is sensitive to temperature. The PDMS [25] used in this experiment was Sylgard-184 produced by Dow Corning, which includes polymer (Sylgard-184-A, referred to as A) and curing agent (Sylgard-184-B, referred to as B). The main component of A is a prepolymer and a small amount of platinum catalyst, while B is a prepolymer with vinyl side chains and a crosslinking agent.

**Figure 4:**
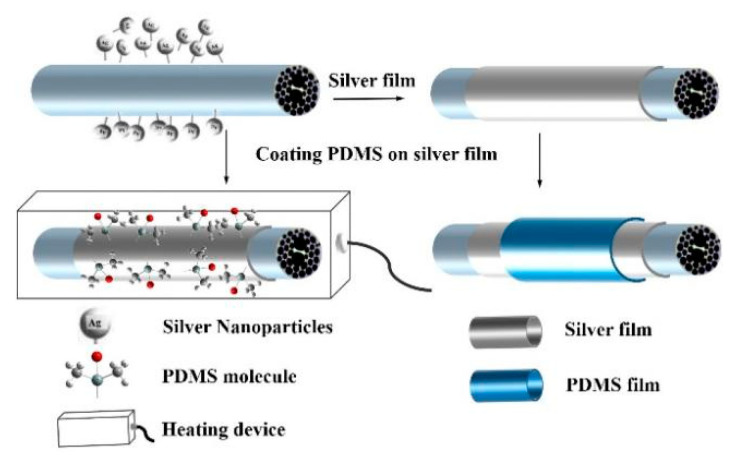
Pouring silver film and PDMS on the sensor and curing the polymer.

The specific operation was as follows: polymer A and curing agent B were mixed in a mass ratio of 10:1 to obtain a uniform colloidal mixture. The colloidal mixture was sputtered uniformly onto the silver film surface of the MSF using a laboratory-made nozzle, and then the solid PDMS film was formed by heating it for 40 min at 100 °C. The thickness of the PDMS film was controlled by sputtering intensity and time [26]. Subsequently, the curing process was used to coat the PDMS on the outside of the sensing part, which effectively reduced the difficulty of the operation and improved the accuracy of the experiment. The three-dimensional structure diagram of the sensing part and the specific production process is shown in Figure 4. Similarly, in this three-dimensional structure, the air hole part is selected as the display part of the cross-section to enhance the readability of the figure.

## 3. Results and Discussion

The model of the sensing structure constructed in this experiment was as follows: MMF-MSF-MMF. The light emitted by the broadband light source (Avantes) was coupled to the microstructured fiber through a multi-mode fiber, and then the output was transmitted to the spectrometer (Ocean Optics USB4000) by means of the multi-mode fiber for detection and analysis. The Ocean Optics USB4000 spectrometer can process spectrum data in real time. The relative measurement value was obtained on the basis of the absolute value identified by the spectrometer to procure quantifiable data results. The relative transmittance is the percentage of the energy passing through the sample relative to the original reference amount. The specific formula is as follows:

(4) $$T_{\lambda }=\frac{S_{\lambda }-D_{\lambda }}{R_{\lambda }-D_{\lambda }}\times 100\%$$

**Figure 5:**
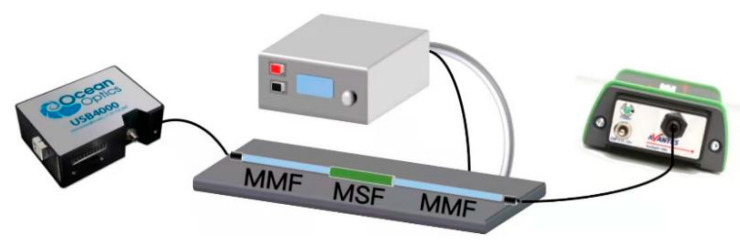
Experimental setup for testing the temperature sensor, including MMF and MSF coated with silver film and PDMS.

In this formula, *S_λ_* is the relative transmission intensity at the wavelength *λ*, *D_λ_* is the background light intensity at the wavelength *λ* and *R_λ_* is the reference light intensity at the wavelength *λ*. Figure 5 displays the experimental device used to test the temperature sensing. The effect of temperature sensing was controlled by a temperature control box (LTD2-250) and a laser chiller (DIC002ASL-LA1).

**Figure 6:**
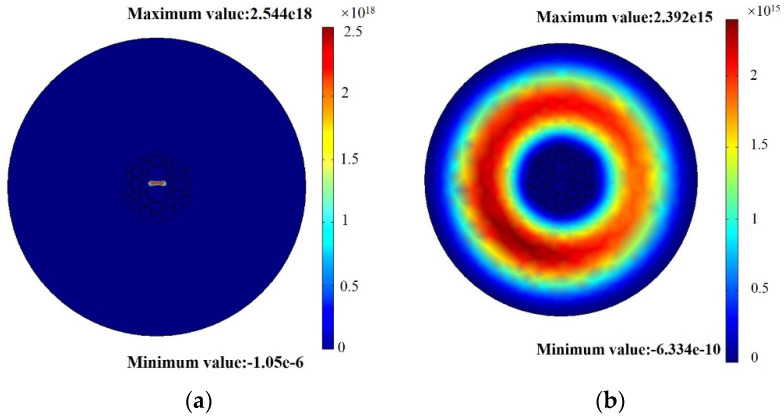
(**a**) The fundamental mode; (**b**) the cladding mode of microstructure fibers.

The theoretical analysis of the feasibility of the experiment was as follows: when the incident light wave enters the MSF, the p-polarized light at the interface is reflected between the outer cladding layer and the silver film. When the thickness of the silver film is less than the thickness of the evanescent wave, the light will pass through the outer cladding and the silver film to reach the junction of the silver film and the temperature-sensitive material, which will stimulate the SPR phenomenon and give full scope to the thermo-optical properties. The MSF cannot only guide light in the core area (Figure 6a), but it also has a mode for guiding light in the outer cladding, which we refer to as the cladding mode, as shown in Figure 6b. This part of the energy will form a high-energy ring in the entirety of the cladding area so that the light energy is more concentrated near the metal layer. Thus, it can excite the SPR more effectively [11,27].

After confirming the theoretical feasibility, the experimental ideas based on this principle were as follows. To study the distinct phenomenon of sensing structure, we sought to determine the optimal length of the microstructure fiber. The selected experimental method was to only change the length of the microstructure optical fiber as the core component while keeping other experimental conditions such as silver plating and welding completely the same. 

**Figure 7:**
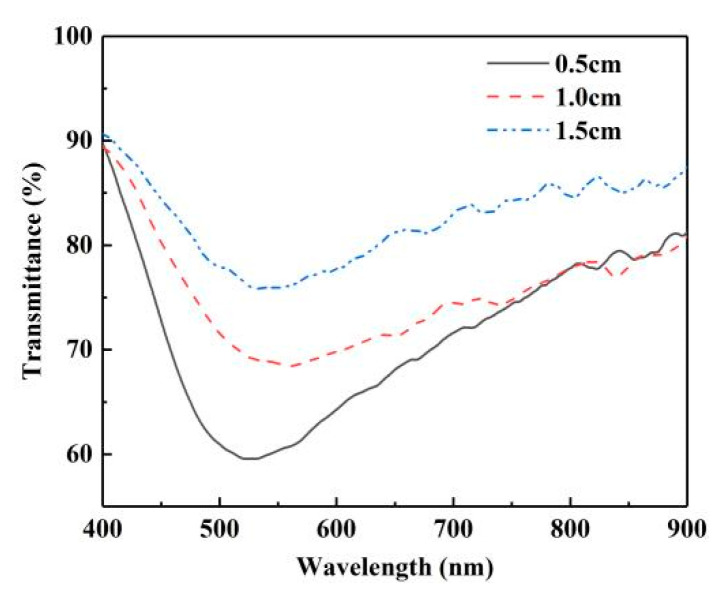
Normalized transmission spectrum for different lengths of MSF.

In the first group, different lengths (0.5 cm, 1.0 cm, 1.5 cm) of MSF were selected to construct the sensor structure under the same silver plating conditions (see Section 2.1) and welding. The welding parameters were as follows: discharge time of 1.5 s, preparation discharge time of 0.05 s and propulsion of 10 µm. Three groups of sensing structures were placed in the solution, with a refractive index of 1.3328. The reference spectrum was the air spectrum of different structures. Because the three groups of structures and reference spectra were both different, we only compared the shapes of spectral lines to determine the appropriate length. Figure 7 shows the normalized transmission spectrum of the three sensors that we fabricated; the three transmission dips are located at 527.8 nm, 553.2 nm and 544.4 nm, respectively. At present, the completion of optical fiber sensor devices pursues sensitivity and high integration, so the purpose of our experiment was to obtain a more significant characteristic spectrum and miniature sensor devices. In consideration of dip width and dip loss, we selected the MSF with a length of 0.5 cm as the optimal length for this sensor from the results shown in Figure 7. All subsequent experiments in the article used the MSF with a length of 0.5 cm, as determined by this group of experiments, to increase the persuasiveness and rigor of the experiment. The welding conditions, solution concentration and reaction time of the silver mirror reaction silver plating film were the same to ensure the greatest degree of control variables.

At the same time, it was also confirmed that the use of the MSF could stimulate the SPR effect and realize refractive index sensing. Since temperature sensing is based on refractive index sensing, subsequent temperature sensing experiments are feasible. Due to the large thermo-optical coefficient (−4.66 × 10^−4^/°C) and large thermal expansion coefficient (9.6 × 10^−4^/°C), we introduced PDMS materials to explore the temperature response. 

**Figure 8:**
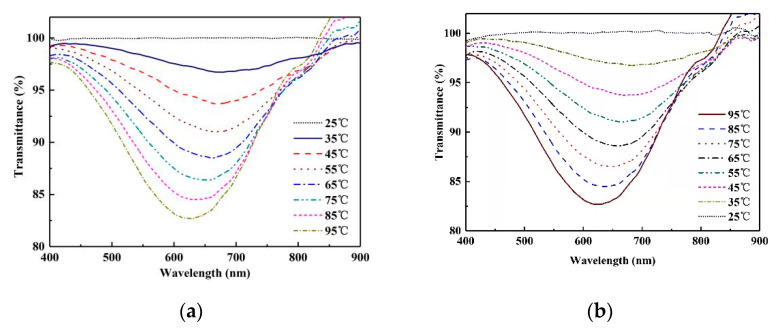
Normalized transmission spectrum of the MSF temperature sensor under: (**a**) the heating process; (**b**) the cooling process.

Subsequently, the temperature sensing characteristics of the MSF coated with PDMS will be discussed. The sensing area of the sensor was coated with PDMS 0.5 cm in length. The spectrum at 25 °C was used as the reference spectrum. When the temperature changed from 35 °C to 95 °C, a stable spectral output was recorded every 10 °C. The processes of heating and cooling were recorded in the experiment, and their normalized transmission spectra are shown in Figure 8a,b, respectively. Attributing the spectral behavior to the negative thermal coefficient of PDMS, it is apparent that the refractive index of PDMS decreases rapidly upon heating, which in turn blue-shifts the resonance spectrum.

**Figure 9:**
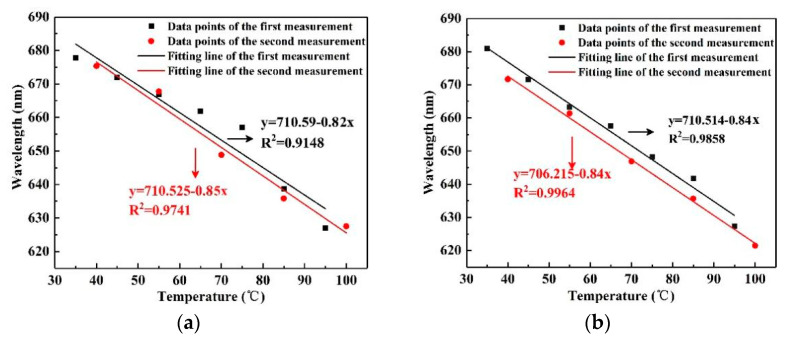
(**a**) The relation spectrum between wavelength and temperature in the heating process of the MSF sensor; (**b**) the relation spectrum between wavelength and temperature in the cooling process of the MSF sensor.

For the analysis of the actual experimental results, we chose to compare the two results with the largest difference in fitting curves from the results of multiple measurements. By carrying out the linear fitting between the resonance wavelength and temperature, we obtained the temperature sensitivity and the linearity of the sensor during heating and cooling, as shown in Figure 9, labeled for the first and second measurements. Figure 9 shows the sensitivity and linearity of the two measurements. Figure 9a is a comparison of the two results at an elevated temperature; the heating sensitivity values were −0.82 nm/°C and −0.85 nm/°C, respectively. Therefore, the average sensitivity of the sensor in the heating process was −0.83 nm/°C. Similarly, the average sensitivity of the cooling process was −0.84 nm/°C, as seen in Figure 9b, based on the results of the two measurements, which were −0.84 nm/°C and −0.84 nm/°C, respectively.

To investigate the experimental stability of the temperature sensor, the relative sensitivity deviation (RSD) was defined as the evaluation index. The calculation formula is RSD = |(S_H_ − S_C_)/(S_H_ + S_C_)|, wherein S_H_ and S_C_ are the average sensitivity values of temperature rising and decreasing, respectively. The smaller the RSD value, the higher the stability. In this experiment, the average heating sensitivity and average cooling sensitivity were calculated. The RSD was calculated at 0.0059. Therefore, the MSF temperature sensor wrapped by PDMS had high repeatability stability.

## 4. Conclusions

In summary, we have demonstrated a simple structure, low cost and good reversibility sensor based on SPR to detect temperature. The sensing part of the sensor is the MSF with a length of 0.5 cm, coated with silver and PDMS films. By analyzing the guided mode of light in the outer cladding layer, the feasibility of using the SPR principle to conduct experiments was determined. After the PDMS layer was wrapped on the silver film, the thermal optical coefficient of PDMS led to a shift in the normalized transmission spectrum as the temperature was varied. The results showed that the heating and cooling temperature sensitivity of the sensor were −0.83 nm/°C and −0.84 nm/°C, respectively. At the same time, the stability of the sensor was investigated by cyclic experiment. The RSD equals 0.0059, which shows that the sensor has adequate stability.

## Data Availability

The data presented in this study are openly available.
